# How Lived Experience Advisory Groups Contribute to the Design and Conduct of Mental Health Research

**DOI:** 10.1111/hex.70722

**Published:** 2026-06-14

**Authors:** Jo Evans, Samuel Keightley, Angela Sweeney, Rosie Hill, Ezekiel Khayri, Sarah Markham, Til Wykes

**Affiliations:** ^1^ Maudsley Biomedical Research Centre King's College London London UK; ^2^ Service User Research Enterprise King's College London London UK; ^3^ Lived Experience Working Group

**Keywords:** impact, lived experience, mental health research, patient and public involvement, research advisory groups

## Abstract

**Introduction:**

The positive impact of patient and public involvement (PPI) on the relevance, accessibility and acceptability of mental health research is well documented. Much of this reporting comes from researchers; less is known about the perspectives of PPI contributors. We are interested in exploring which issues people with lived experience (LE) prioritise when reviewing mental health research, with a view to identifying some of the underlying factors which facilitate or hinder those impacts.

**Methods:**

We conducted a reflexive thematic analysis of meeting minutes reviewing 233 studies from two PPI research advisory groups supported by the Maudsley Biomedical Research Centre over a period of 8 years.

**Results:**

Our results foreground participants' experiences and show that PPI contributors want a greater emphasis on relational ethics within mental health research practice. We have identified key issues that have the potential to have a positive effect on the whole research cycle. These include ensuring fully inclusive research designs and practices, having transparent processes and increasing researcher reflexivity regarding power dynamics, stigma and participants' experiences.

**Conclusion:**

This study demonstrates the potential that PPI has to supplement existing ethical processes and positively impact research processes.

**Patient or Public Contribution:**

People with lived experience of mental distress were involved throughout the study, including study design, data collection, synthesis and authorship.

## 1 Introduction

It is increasingly accepted that patient and public involvement (PPI) improves the relevance, accessibility and acceptability of health research [1, 2]. PPI practice is encouraged by major funders and some journals, as well as the recently updated CONSORT and SPIRIT statements, which all prioritise the inclusion and reporting of PPI in research [3, 4, 5, 6, 7]. The impact of PPI is well‐documented and includes a positive influence over research priorities and research questions, recruitment and retention, data collection processes and data interpretation. Researchers gain insight, as well as improved communication and collaboration skills and at a wider level, PPI can alleviate power imbalances and challenge stigma, although this is not always successful [8, 9, 10, 11, 12, 13].

Despite these benefits, sometimes PPI can be performative and tokenistic, particularly within academia [14, 15]. Implementation tends to reflect a technocratic approach, which aims to improve the quality, relevance and utility of research by focusing on processes and outputs [16, 17, 18]. Visible, easily measurable impacts are the ones most valued by researchers and therefore most frequently reported [19]. These aims are important but considerably less focus is placed on how PPI can support research practices that centralise participants' wellbeing and autonomy [20]. This is important because whilst there are many benefits to research participation [21], there can also be psychological, physical and financial costs, sometimes with little direct benefit from the research outcome itself [22, 23, 24]. Procedural ethics, in the form of research ethics boards, have an established safeguarding role. However, increasing attention is being paid to relational ethics, which prioritises researcher‐participant interaction and promotes empathy, respect, sensitivity and cultural awareness [25, 26, 27].

Successful involvement is dependent on context. Substantial barriers exist within mental health research environments that reinforce the status quo and hinder the potential for change. These include significant power differentials, stigma, the low status placed on experiential knowledge and a lack of diverse representation [28, 29, 30, 31]. Survivor researchers have long argued for an emancipatory approach to involvement, one where power inequalities and epistemic injustice are challenged and research is based on the principles of inclusion and equality [32, 33, 34].

Despite the substantial evidence on the positive impact of PPI on distinct research processes, we know little about which factors facilitate or hinder success. Much of what is reported comes from the perspectives of researchers; less is known from the perspective of PPI contributors themselves. In this study, we extend this evidence by exploring such perspectives and by highlighting the ways in which people with lived experience (LE) can influence the quality of mental health research. To do this, we need a large body of data encompassing a wide range of research studies. This was provided by the meeting minutes of two PPI research advisory groups over a period of 8 years.

## 2 Methods

### 2.1 Setting

This study is set within the NIHR Maudsley Biomedical Research Centre (BRC), a collaboration between the South London and Maudsley NHS Foundation Trust and King's College London, which aims to improve the translation of research into practice. The Maudsley BRC hosts two regular PPI research advisory groups: the Service User Advisory Group (SUAG) and the Young People's Mental Health Advisory Group (YPMHAG), established in 2009 and 2014 respectively. All group members have direct lived experience of mental distress or of caring for people with such experience, as well as an interest in mental health research. On joining, they receive basic training on research ethics and design.

Researchers are asked to provide a brief lay summary in advance, that is included in the agenda so members can consider the study before the meeting. At meetings, researchers present their study, followed by a discussion and feedback from the group. The group facilitators send presenters a feedback summary and send the meeting minutes to members, providing them with an opportunity for correction. Meeting frequencies and structure differ by group. The SUAG currently meets bimonthly and the YPMHAG every quarter, although the frequency varied in previous years. SUAG meetings last for 90 min and cover two 30‐min research presentations, whereas YPMHAG meetings last for 4 h, including three 45‐min presentations and a debrief with members. Both groups met in‐person until the onset of the Covid‐19 pandemic, at which point meetings transitioned online. Since restrictions lifted, the SUAG remains predominantly online whereas the YPMHAG has a mixture of online and in‐person meetings.

During the timeframe of this study, the SUAG was facilitated by the lead author and the YPMHAG by a succession of facilitators. SUAG attendance ranged from 12 to 15 people, with an average of seven members per meeting and for the YPMHAG, ranged from 9 to 26 people, with an average of twelve per meeting. SUAG membership was open to all ages, however members tended to be older and white and membership remained relatively constant. The YPMHAG was established as a group for 16‐25 year olds, was more ethnically diverse and the membership evolved as people aged out of the group. To preserve anonymity, we have chosen not to provide specific demographic data for members.

### 2.2 Study Data

The study data comprises 106 meeting minutes (SUAG = 52, YPMHAG = 54), reviewing 233 studies over 8 years, 2017‐2024. The minutes showed that the structured content of the meetings differed, depending on the group and facilitators. For example, the SUAG meetings were summarised by theme, whereas the YPMHAG minutes included examples of member's experiences of mental distress and services to illustrate points. The minutes varied in length (one to four pages), depending on the level of discussion and feedback. They included the lay summary of the study, followed by the group's feedback.

### 2.3 Theoretical Approach

We employed a critical realist ontology and contextualist epistemology. These positions assert that experiences are context‐driven [35] and that multiple accounts of reality are possible. Within this approach, researchers' values and practices shape the knowledge they produce and therefore reflexivity and transparency are crucial [36]. This study takes a survivor research standpoint, which centralises the direct experience and identity of people who have used mental health services [37].

### 2.4 Data Analysis

**Figure 1:**
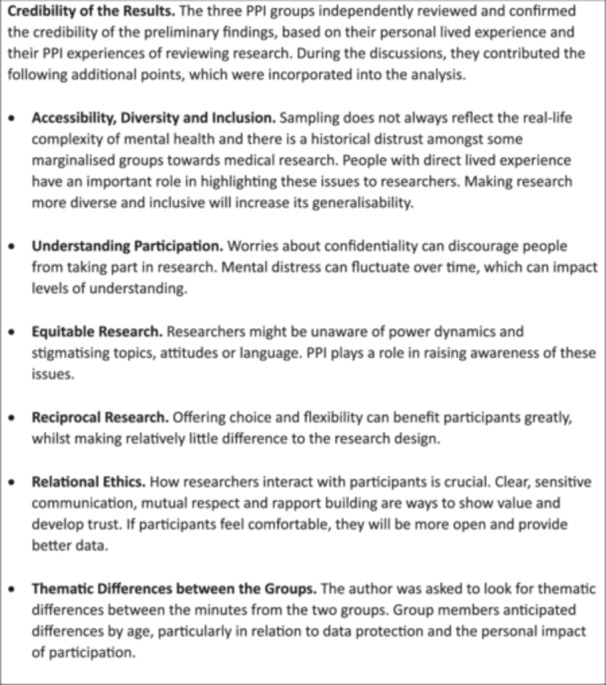
Contributions from the PPI groups during the analysis validation process.

**Figure 2:**
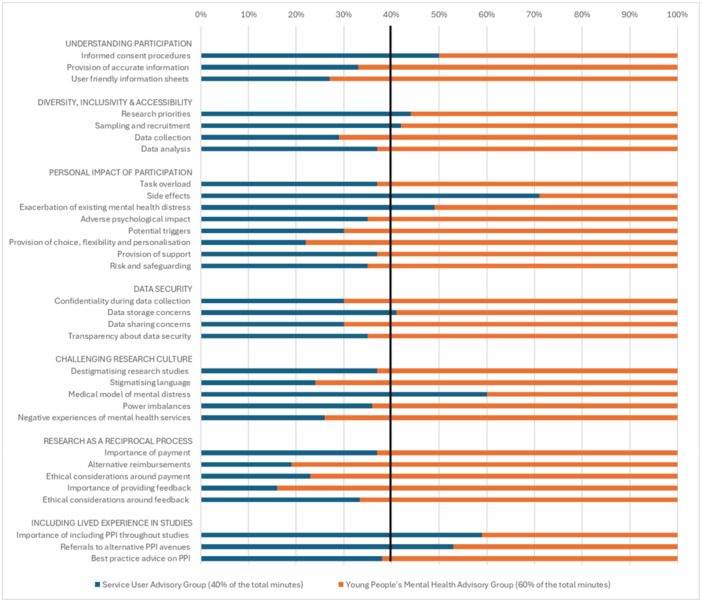
Themes identified within the group minutes by percentage.

The two sets of advisory group minutes were qualitatively analysed to explore which issues were prioritised by members. Data were collated on the research area, design and method. We undertook a reflexive thematic analysis [36] using NVIVO14 and as the research question was exploratory in nature, we used an inductive approach. After data familiarisation, two researchers went through the data line‐by‐line to generate initial codes, which were then grouped into descriptive themes within a hierarchical coding frame. Through iterative discussions, we refined the coding structure, resolved discrepancies and developed broader analytical themes by identifying patterns and relationships across the data. The coding frame and thematic structure were continually reviewed and adjusted to maintain consistency throughout the analysis. The initial findings were then presented to the SUAG, YPMHAG and an independent lived experience PPI group to check the credibility of our interpretation (Figure 1) [38]. We anticipated there may be thematic differences between the two groups because of the age difference, therefore we conducted a qualitative content analysis of the dataset (Figure 2) [39].

### 2.5 Ethical Considerations

The study received approval by the Research Ethics Committee of King's College London (LRS/DP‐22/23/36853). As all minutes were already anonymised, formal consent from group members and attendees was not required. However, we did reach out to group members and presenters to outline the purpose and nature of the research. We wanted to protect the confidentiality and anonymity of everyone concerned and made great efforts to ensure that any examples or quotations given could not be traced back to specific members or presenters. We have also chosen to report an overview of the research studies. Again, the information has been anonymised and collated, rather than reported individually.

### 2.6 The Involvement of Experiential Knowledge

People with lived experience of mental distress were involved throughout the study, including study design, data collection, synthesis and authorship. The lead author, an LE researcher, collaborated with a Working Group of people with lived experience or being a caregiver. The six members of this group were drawn from existing PPI advisory groups within the Maudsley BRC, including two each from the SUAG and YPMHAG. All three groups independently contributed to the validation of the findings (Figure 1), and some are authors of this manuscript.

## 3 Results

One hundred and six sets of meeting minutes were included, reviewing 233 studies and covering the development of interventions, therapies, medications, novel methodologies and the identification of risk factors. See Table S1 for details.

### 3.1 Reflexive Thematic Analysis

**Figure 3:**
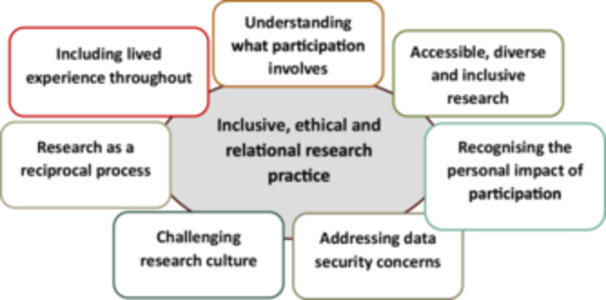
Thematic analysis summary.

Members were generous in sharing their personal experiences of distress and services to illuminate and provide context to the feedback given to researchers. The minutes showed that members were overwhelmingly positive about the potential impact of the studies they reviewed. They believed them to be important and valuable and felt they would improve the evidence base, NHS services and the lives of people with lived experience. In addition, members acted as knowledge providers, frequently signposting researchers to alternative resources to inform their research. However, the minutes also highlighted some key issues, which members felt were less considered by researchers. See Figure 3 for an overview of the thematic analysis and Table S2 for an overview of the themes by research stage (see supporting material). The three PPI groups independently reviewed and confirmed the credibility of the preliminary findings, based on their personal lived experience and their PPI experiences of reviewing research. During the discussions, they contributed additional insights, which were incorporated into the analysis (see Figure 1).

### 3.2 Understanding What Participation Involves

The importance of informed consent was highlighted consistently by members. Many queried the processes involved, especially where capacity was concerned.

Is the child old enough to understand what is involved and the implications, given the long‐lasting effects?

(S2, 2023)^1^

Intrinsic to this process was the provision of full and accurate information for participants. Members wanted this in the form of user‐friendly information sheets and consent forms, including imagery and brief, accessible, clear and sensitive language.

The complexity of the language varies a lot in the document. It would be helpful to find simpler ways to explain this study.

(S56, 2019)

They felt these documents should include the study aim and benefits, the tasks involved, information on risk, safeguarding and support and the voluntary nature of participation, as well as reassurances over data confidentiality. However, members cautioned against the provision of information as a one‐off event. Instead, they recommended ongoing informal discussions to aid understanding for participants throughout the research process.

### 3.3 Ensuring Research Is Accessible, Diverse and Inclusive

Ensuring diversity, inclusion and the accessibility of research were high priorities and cut across all stages of the research process, but particularly the need for more inclusive and relevant research priorities and more sample diversity to increase the generalisability of research.

You're excluding people with insufficient English. Would you be able to use interpreters so your sample is more diverse?

(S100, 2024)

During recruitment, members identified barriers that might be faced by some communities and emphasised the need for welcoming, user‐friendly advertising, the use of alternative recruitment methods and the need for researchers to make greater efforts to reach out to find participants.

Text heavy, especially if recruiting for individuals with ADHD.

(Y80, 2019)

Members drew attention to inaccessible data collection processes and made recommendations such as providing interpreters and translated materials, using diverse and gender‐neutral imagery, payment of travel expenses, adapted measures for neurodiverse participants and online options for people with disabilities.

Even pre‐pandemic, there was a push from disabled groups to have meetings online and Covid has taught us it is possible. It is nice to do it in‐person, but some people can't travel.

(Y44, 2021)

They also highlighted the potential for digital exclusion, which could be more marked for those on lower incomes. In terms of data analysis, members provided context based on their lived experience and emphasised the influence of intersectionality on data interpretation.

### 3.4 Recognising the Personal Impact of Participation

One of the largest themes raised by members was the impact of data collection on participants, particularly task overload.

The group felt that ten questionnaires a day over a 1‐year period may be unfeasible and that participants would not maintain this level of responding beyond 1 week.

(Y120, 2017)

There was concern that existing mental health distress could be exacerbated by task requirements, the subject matter and the language and imagery used. Members also felt participants could be affected by power imbalances, being observed or measured, interactions with researchers and a lack of privacy. They highlighted the potential for anxiety, discomfort and distress, fear and disempowerment. The side effects of medication, especially clozapine, ketamine and psilocybin, were commonly cited.

It is not sufficient to screen out participants with a history of psychosis as some people are unaware that this may be a risk factor for them. This has the potential to trigger a first episode of psychosis, which is a frightening and dangerous experience. The safeguards in this study seem good but they don't prevent risk.

(S17, 2022)

To support data collection, members suggested that researchers provide choice, flexibility and personalisation where possible, offer advance preparation and to make efforts to put participants at ease and support them with tasks. They also encouraged the use of chaperones, breaks and additional check‐ins. Crucially, members raised the importance of monitoring, evaluating and responding to risk when it occurred, as well as signposting to appropriate support and safeguarding.

I think in terms of how they do their interview/who they want there, it's always best to let people choose what makes them most comfortable.

(Y32, 2021)

### 3.5 Addressing Data Security Concerns

Data management was a frequent concern for members. Privacy and confidentiality during data collection were paramount, often during focus groups when sensitive subjects such as self‐harm were discussed or when a child's parents were present. Members asked whether data storage was secure, how long data would be retained and how it would be used. Data sharing was a large concern, specifically whether information would be shared with care teams or school staff and how this might affect care. Members felt that these concerns could inhibit recruitment and limit disclosure during data collection.

People may not accurately report their feelings with regards to their physical and mental health for fear of worrying the researcher and their parents/carers being informed. Therefore, the issue of confidentiality must be considered and explained.

(Y129, 2017)

Members felt transparency at the outset was important. They wanted researchers to provide clear information, in lay language and accessible formats, on why data was being collected, how it was anonymised, used and securely stored. This information should also include the circumstances in which confidentiality could be broken and reassurances that participation would not affect care.

I would want to know GDPR/privacy policy, clearly who would be seeing info and that it wouldn't be used against participants. Assurance it won't come back later.

(Y13, 2022)

### 3.6 Challenging Research Culture

The minutes demonstrated that members played a significant role in raising awareness, challenging assumptions and encouraging researchers to reflect on their attitudes and working practices. Members felt that stigma was commonly experienced by people living with mental distress, especially those from minoritised communities. They highlighted issues with some studies and study materials, and suggested ways to destigmatise practice. Language was of particular concern as it was seen to reflect medical models of mental health and a lack of understanding and respect.

The term ‘delusions’ is not helpful. Please be aware that these experiences are real to the people having them and calling them delusions can feel dismissive and labels them.

(S48, 2019)

Members also asked researchers to consider the influence of power imbalances within psychiatric research. This was linked to negative, disempowering experiences of mental health services and often related to minoritised communities.

With online workshops, will that be together for professionals and service users? For people with lived experience this might be intimidating, the language used is different and the power balance is a concern (agreed by other members). People might not be as truthful in that setting.

(Y19, 2022)

### 3.7 Establishing Research as a Reciprocal Process

Members stressed the importance of paying participants adequately, including travel expenses, for their time and contributions to studies. As well as cash, they suggested alternative reimbursements such as vouchers, copies of brain scans or wearables. They did raise some ethical concerns, such as the potential for payment to be construed as pressure to take part and how best to compensate child participants, as well as emphasising the need for clarity on payment at the outset of studies.

Some kind of compensation for time and talking about potentially triggering emotional subjects is good.

(Y42, 2021)

Providing feedback was also seen as integral to the research process, including regular progress updates and final results in clear, accessible formats. They saw this as a way to increase long term engagement in studies and show value for participants. Members also suggested giving participants personalised updates on individual test results. However, they had concerns that this might generate anxiety, especially if support was not available. For all feedback, using sensitive language and giving participants the choice of whether to opt‐in to receiving information were important.

It may be particularly valuable to get immediate feedback. However, they suggested this could be viewed as too much/emotionally draining after discussing the physical and mental difficulties they are experiencing. The group suggested that asking for feedback a few days/the following week may be better received.

(Y129, 2017)

### 3.8 Including Lived Experience in Research

Members were interested in determining the levels of PPI within studies, as well as recommending that researchers add PPI from the outset and throughout the research cycle. They also referred researchers to alternative PPI avenues for advice and support.

It's really important to get PPI throughout as much of the study as possible so that you can resolve any issues/obstacles as they arise.

(S108, 2024)

Members made numerous suggestions on best practice, including the recruitment and long‐term engagement of PPI contributors and the use of study PPI advisory groups and stressed the importance of regular contact and communication, support and payment.

I think they should be paid like any other job. They are giving up their time.

(Y38, 2021)

They highlighted how gaining and providing feedback to contributors made them feel heard and valued.

### 3.9 Thematic Differences **B**etween the Groups

A qualitative content analysis showed some differences in themes between the two groups (see Figure 2). The YPMHAG tended to focus on practical issues within the research process, while the SUAG took a wider perspective. For example, SUAG members focused on the process of gaining consent and the existence of PPI within studies, whereas YPMHAG members suggested changes to information sheets to make them more user‐friendly.

Clearly explaining exactly what the study will involve will be helpful, and visualisations could be useful for both parents and children/young people.

(Y98, 2018)

Secondly, YPMHAG members placed a greater emphasis on ensuring that research was a reciprocal process. In terms of data collection processes, the SUAG were particularly concerned about side effects, whilst the YPMHAG highlighted the potential for upsetting language and imagery and emphasised the importance of providing choice, flexibility and personalisation.

Researchers should be mindful of terminology that can be triggering.

(Y62, 2020)

Raising researchers' awareness of stigma was important to both groups. SUAG members highlighted the presence of a medical model of mental health, whereas YPMHAG members called attention to stigmatising language.

There's a concern that ADHD is being seen as a brain defect that needs to be treated, rather than as an aspect of neurodiversity. It implies that ADHD brains should be artificially altered to fit into the neurotypical world.

(S2, 2023)

Finally, YPMHAG members were more likely to highlight concerns with data security, especially confidentiality during data collection and data sharing.

## 4 Discussion

In exploring the priorities of advisory group members, our study has identified ways in which PPI can positively impact mental health research. We have illustrated how people with lived experience prioritise full and equitable involvement in the production of knowledge. This is both through the co‐design and conduct of research and by centralising participants' experiences, prioritising their autonomy and well‐being to ensure richer data and more relevant research outcomes. This focus on relational ethics is largely missing from the impact literature [8, 9, 10, 11, 12, 13]. Issues raised include ensuring fully inclusive research designs and practices, having transparent processes and increasing researcher reflexivity regarding power dynamics, stigma and participants' experiences. This reflects the principles of a more democratic model of involvement, as argued for by survivor researchers [32, 33, 34]. Although these findings may not be applicable to all PPI practice nationally, they provide valuable knowledge which is transferable to similar contexts, particularly within academia.

### 4.1 Implications for Research Practice

Certain groups are under‐represented within mental health research [40], and PPI plays a key role in highlighting this to researchers. This study has shown that people with lived experience want research design and conduct to be more widely inclusive, representative and accessible so that it becomes more relevant to their needs. This is essential when considering groups who have been traditionally disadvantaged within psychiatry or those with a historical distrust in services [15, 41]. Mental health research should not replicate systemic inequalities but aim for more inclusive practices, with associated potential benefits to engagement, research relevance and generalisability. The adoption of these practices is growing, driven by initiatives like the Health Research Authority's Inclusion and Diversity Plans, which provide a valuable framework for researchers [42].

A key component of inclusion and accessibility is the provision of simple, clear information to aid participants' understanding. Recent research shows that, despite ethical approval, a relatively low number of participants feel fully prepared by the information and consenting process [43]. For many, complex research processes can be confusing and language, especially the use of jargon, can be alienating and hinder understanding. A lack of transparency around data protection or concerns about confidentiality during data collection can also be off‐putting. Our results confirm the importance of clarity and transparency throughout research processes to ensure that participants fully understand what their involvement entails [22]. They also suggest the need for ongoing, informal discussions to ensure continued understanding throughout the research process. This is particularly important, given the fluctuating nature of mental distress. Focusing on these issues may positively impact recruitment and retention and encourage honesty during data collection, thereby yielding higher quality data.

At the heart of ethical practice are equitable research environments, where everyone involved feels respected and supported. Consistent with previous research [15, 28, 30, 31], group members highlighted the presence of power hierarchies and stigma within mental health research. These dynamics are not always readily apparent to researchers and PPI plays a significant role in heightening their visibility. We concur with Russell, Fudge & Greenhalgh [19] that impact should be considered not only in terms of measurable outcomes but also changes in power and relationships. Encouraging researchers to reflect on their assumptions and address stigma, biases, language and power imbalances has the potential to lead to more inclusive, respectful and equitable research practices [20, 44], which may in turn increase credibility and trust in research.

Equally important was the belief that research should become a more reciprocal process, one from which both researchers and participants benefit. In addition to payment, the groups emphasised the importance of feeding results back to participants, so they are more invested in the research. Recent studies confirm that this is a priority, yet it is not routine practice [43, 45]. Our members confirmed the vital role that ethics processes play in safeguarding. However, they called for greater attention to participants' needs and provision of support on an ongoing basis. This corroborates a recent review highlighting the adverse psychological, physical and financial costs of research participation [22]. Reducing the risk of harm or distress during data collection is paramount, particularly given the sensitive and intersectional nature of mental distress [15, 44]. Providing flexibility, where pragmatically possible, in appointment times and modes of data collection, and increasing communication and rapport can aid this process and help to ensure studies are more sensitive to participants' needs [43, 46]. Creating research environments where participants feel safe, valued and supported may in turn improve engagement, generate richer data and enhance research credibility at a wider level.

Research ethics committees play a vital role in safeguarding the rights, safety and well‐being of research participants [47] and our data confirms that these are also priorities for people with lived experience. Ethics committees are concerned with procedural ethics such as regulation compliance, structured risk management and documented consent. Where our PPI contributors differ is in placing more of an emphasis on relational ethics; ensuring that research is relevant, accessible and understandable to people with lived experience and that where possible, interactions between researchers and participants become more flexible and reciprocal. The data in this study are drawn from one‐off PPI consultation processes. Increasing PPI and co‐production within research studies from the outset will play a vital role in addressing these issues, as well as aiding the process of ethical approval [48]. Increasing LE representation on ethics committees is also recommended so that the people most affected by the research are involved in decision making about its conduct [49, 50, 51].

It was apparent that there were some thematic differences in the minutes from the two groups. This may have been due to different studies being reviewed by the different groups. It may also, in part, have been influenced by the priorities of the researchers presenting to the groups. For example, the SUAG was more likely to be asked to focus on study design, whereas the YPMHAG was asked for more practical advice on engaging participants and the accessibility of study materials. The difference in emphasis may also be due to the age differences between the two groups. The younger YPMHAG members called for greater attention to the emotional impact of participation and data security issues, indicating a heightened concern for younger participants' vulnerability and their levels of understanding of research processes. These findings echo a recent review of adolescents' experiences of participation and emphasise the importance of reducing power dynamics, ensuring robust consent procedures and providing support and safeguarding in mental health research with young people [52, 53].

### 4.2 Research Team Reflexivity

In any survivor research, people with lived experience of mental distress must be involved from the outset and throughout. As a core research team, we are predominantly white, older and cisgender. Some researchers have direct lived experience, some do not, and there are varying levels of understanding around PPI. We were reflexive about these influences and widened our perspectives through collaboration with PPI groups, who played a prominent role in data validation. We believe that this anchors the findings and increases their relevance to researchers, clinicians and those receiving services. Integration of different perspectives, especially during data analysis, was sometimes challenging and required strong collaboration and communication skills. However, we contend that the diversity of perspectives strengthened and enriched our research processes and outcomes [54, 55].

Working Group members were drawn from existing PPI advisory groups. We felt they would add value to the study because of their direct knowledge and experience of volunteering within the organisation under study. Two were also SUAG members and therefore had a working relationship with the lead author. To mediate this, we reflected on the potential influence this familiarity may have had and acted to reduce any power dynamics that might be at play. We also recruited members unknown to the team, encouraged openness and worked to ensure everyone had an equal voice.

### 4.3 Study Limitations

A limitation, raised by the SUAG during the data synthesis session, arises from the way that meeting minutes were recorded. One facilitator, the lead author, produced the SUAG minutes, whereas the YPMHAG minutes were produced by several different facilitators. Minute taking also evolved over the 8 years of recording and both these factors may have affected summarisation and subsequently data interpretation. However, many similar themes were apparent between the groups, suggesting that these differences did not have a major influence.

## 5 Conclusion

PPI research advisory groups provide a valuable opportunity for reflexivity between researchers and people with lived experience. Our results foreground participants' experiences and show that PPI contributors want a greater emphasis on relational ethics within mental health research practice. This study demonstrates the potential that PPI has to supplement existing ethical processes and positively impact research processes.

## Author Contributions

**Jo Evans:** conceptualisation, methodology, investigation, writing – original draft, writing – review and editing, formal analysis, project administration, data curation, software, validation. **Samuel Keightley:** writing – review and editing, formal analysis. **Angela Sweeney:** writing – review and editing, project administration, supervision. **Rosie Hill:** validation, writing – review and editing. **Ezekiel Khayri:** Validation, writing – review and editing. **Sarah Markham:** validation, writing – review and editing. **Til Wykes:** funding acquisition, writing – review and editing, project administration, supervision.

## Ethics Statement

The study received approval by the Research Ethics Committee of King's College London (ref number LRS/DP‐22/23‐36853).

## Conflicts of Interest

The authors declare no conflicts of interest.

## Supporting information

Supporting File 1

Supporting File 2

## Data Availability

The data that support the findings of this study are available on request from the corresponding author. The data are not publicly available due to privacy or ethical restrictions.

## Endnotes

1 S = SUAG, Y = YPMHAG.
