# Supplementary material for: How Lived Experience Advisory Groups Contribute to the Design and Conduct of Mental Health Research
Source: Health Expect. 2026 Jun 14;29(3):e70722. doi: 10.1111/hex.70722 (PMC13264677; doi:10.1111/hex.70722)
Supplement: Supplementary file 1 — Supporting File 1 [file HEX-29-e70722-s001.docx]

**Table S1: Meeting minutes (n=106) with reviews of research studies (n=233)**

|  | **No. of studies reviewed (%)** |
| --- | --- |
| **Advisory Group** |  |
| Service User Advisory Group (SUAG) | 94 (40) |
| Young People’s Mental Health Advisory Group (YPMHAG) | 139 (60) |
|  |  |
| **Research Area** |  |
| General mental distress | 79 (34) |
| Depression and/or anxiety | 45 (19) |
| Schizophrenia and/or psychosis | 36 (16) |
| Eating distress | 12 (5) |
| Neurodiversity | 12 (5) |
| Comorbidity | 5 (2) |
| Other | 44 (19) |
|  |  |
| **Design** |  |
| Observational | 154 (66) |
| Experimental | 79 (34) |
|  |  |
| **Methodology** |  |
| Quantitative | 163 (70) |
| Qualitative | 41 (18) |
| Mixed | 29 (12) |
