# Supplementary material for: How Lived Experience Advisory Groups Contribute to the Design and Conduct of Mental Health Research
Source: Health Expect. 2026 Jun 14;29(3):e70722. doi: 10.1111/hex.70722 (PMC13264677; doi:10.1111/hex.70722)
Supplement: Supplementary file 2 — Supporting File 2 [file HEX-29-e70722-s002.docx]

**Table S2: Research themes by research stage**

|  | Research Priorities | Recruitment & Retention | Data Collection | Data Analysis | Dissemination |
| --- | --- | --- | --- | --- | --- |
| Understanding what participation involves |  |  |  |  |  |
| Ensuring research is accessible, diverse and inclusive |  |  |  |  |  |
| Recognising the personal impact of participation |  |  |  |  |  |
| Addressing data security concerns |  |  |  |  |  |
| Challenging research culture |  |  |  |  |  |
| Establishing research as a reciprocal process |  |  |  |  |  |
| Including lived experience in research |  |  |  |  |  |
